# Guanine inhibits the growth of human glioma and melanoma cell lines by interacting with GPR23

**DOI:** 10.3389/fphar.2022.970891

**Published:** 2022-09-19

**Authors:** Roberta Garozzo, Mariachiara Zuccarini, Patricia Giuliani, Valentina Di Liberto, Giuseppa Mudò, Francesco Caciagli, Renata Ciccarelli, Francisco Ciruela, Patrizia Di Iorio, Daniele F. Condorelli

**Affiliations:** ^1^ Department of Biomedical and Biotechnological, Section of Medical Biochemistry, University of Catania, Catania, Italy; ^2^ Department of Medical, Oral and Biotechnological Sciences, Section of Pharmacology and Toxicology, School of Medicine, University of Chieti-Pescara, Chieti, Italy; ^3^ Center for Advanced Studies and Technologies (CAST), University of Chieti-Pescara, Chieti, Italy; ^4^ Department of Biomedicine, Neuroscience and Advanced Diagnostic, University of Palermo, Palermo, Italy; ^5^ Pharmacology Unit, Department of Pathology and Experimental Therapeutics, Faculty of Medicine and Health Sciences, Institute of Neurosciences, University of Barcelona, Barcelona, Spain; ^6^ Neuropharmacology and Pain Group, Neuroscience Program, Institut d’Investigació Biomèdica de Bellvitge, IDIBELL, Barcelona, Spain

**Keywords:** guanine-based purines (GBPs), guanine (GUA), purine nucleoside phosphorylase (PNP), antiproliferative effects, G protein-coupled receptor 23 (GPR23), glioma cell lines, melanoma cell lines, lysophosphatidic acid (LPA)

## Abstract

Guanine-based purines (GBPs) exert numerous biological effects at the central nervous system through putative membrane receptors, the existence of which is still elusive. To shed light on this question, we screened orphan and poorly characterized G protein-coupled receptors (GPRs), selecting those that showed a high purinoreceptor similarity and were expressed in glioma cells, where GBPs exerted a powerful antiproliferative effect. Of the GPRs chosen, only the silencing of GPR23, also known as lysophosphatidic acid (LPA) 4 receptor, counteracted GBP-induced growth inhibition in U87 cells. Guanine (GUA) was the most potent compound behind the GPR23-mediated effect, acting as the endpoint effector of GBP antiproliferative effects. Accordingly, cells stably expressing GPR23 showed increased sensitivity to GUA. Furthermore, while GPR23 expression was low in a hypoxanthine-guanine phosphoribosyl-transferase (HGPRT)-mutated melanoma cell line showing poor sensitivity to GBPs, and in HGPRT-silenced glioma cells, GPR23-induced expression in both cell types rescued GUA-mediated cell growth inhibition. Finally, binding experiments using [^3^H]-GUA and U87 cell membranes revealed the existence of a selective GUA binding (K_D_ = 29.44 ± 4.07 nM; Bmax 1.007 ± 0.035 pmol/mg prot) likely to GPR23. Overall, these data suggest GPR23 involvement in modulating responses to GUA in tumor cell lines, although further research needs to verify whether this receptor mediates other GUA effects.

## Introduction

While adenine-based purines (ABPs), including adenosine triphosphate, diphosphate, and monophosphate (ATP, ADP, and AMP, respectively), and adenosine (ADO), have been extensively studied as extracellular signalling molecules in the central nervous system (CNS) (Burnstock, 2018; Jacobson et al., 2020; Illes et al., 2021), the guanine-based purines (GBPs), comprising guanosine triphosphate and monophosphate (GTP and GMP, respectively), guanosine (GUO), and guanine (GUA) has attracted less interest, although they also display CNS effects. Indeed, numerous studies have shown that GBPs are effective neuroprotective agents as they contribute to nervous tissue repair upon brain injury mostly by preventing glutamate excitotoxicity (reviewed in Di Liberto et al., 2016; Mancinelli et al., 2020; Massari et al., 2021; Di Iorio et al., 2021). Furthermore, they exert antiparkinsonian, anticonvulsant, antidepressant, and anxiolytic/amnesic effects (Di Iorio et al., 2021), which have mostly been attributed to GUO. Interestingly, GUA, the nucleobase resulting from purine nucleoside phosphorylase (PNP)-mediated GUO metabolism, exhibited activity in the CNS different from that of GUO, as GUA improved learning and memory formation in behaving animals (Giuliani et al., 2012; Zuccarini et al., 2018). In addition, GBPs stimulate the proliferation of neural stem cells and astrocytes (Ciccarelli et al., 2000; Su et al., 2013) as well as promote the differentiation of neuroblastoma cells or C2Cl2 skeletal muscle cells (Mancinelli et al., 2012; Belluardo et al., 2021). However, in some tumoral cell lines GBPs, in particular GUA, showed anti-proliferative effects mainly linked to an S-phase cell cycle arrest (Garozzo et al., 2010).

The lack of reliable plasma membrane receptors to account for the multiple though sometimes conflicting effects induced by GBPs in the CNS has historically reduced the interest of the scientific community, thus substantially diminishing the study of the guaninergic signalling. So far, a high affinity binding site for [^3^H]-GUO on rat brain has been described (Traversa et al., 2002). In addition, several G-protein-coupled GUO-mediated effects involving cyclic nucleotides and mitogen activated protein kinase (MAPK) and/or phosphoinositol-3-kinase (PI3K)/Akt pathways have been reported (Gysbers and Rathbone, 1992; Di Iorio et al., 2004; Molz et al., 2011; Giuliani et al., 2015). Interestingly, it has been postulated that adenosine A_1_ and A_2A_ receptors (i.e., A_1_R and A_2A_R, respectively) might be somehow involved in GUO-mediated neuroprotection by the stimulation of A_1_R/A_2A_R heteromers (Lanznaster et al., 2019). GTP also seems to harbour specific binding sites, likely G_αi/o_ protein-coupled receptors, in mouse skeletal muscle cells (Pietrangelo et al., 2002). Finally, although the antiproliferative effect of GUA cited above has been referred to an intracellular metabolism of GUA (Garozzo et al., 2010), following the discovery of receptors sensing nucleobases such as adenine (Bender et al., 2002; Gorzalka et al., 2005) it has been reported that isolated basolateral membranes of renal proximal tubule express a G_αi/o_ protein-coupled receptor for GUA, whose stimulation inhibits the activity of Na^+^-ATPase (Wengert et al., 2011).

Altogether, these findings point towards the possible existence of plasma membrane GBP receptors mediating the physiological effects associated to this important group of purines. Accordingly, we aimed to conduct a purinoreceptor homology-based screening within the group of orphan and poorly characterized G protein-coupled receptors (GPCRs) to pinpoint those responding to GBPs. Our bioinformatic approach revealed some potential candidates within these GPCRs, which were subsequently studied in some cancer cell lines previously used for assessing GBP functionality (Garozzo et al., 2010).

## Materials and methods

### Chemical compounds

GUA, GUO, GMP, GTP and ADO were purchased from Sigma-Aldrich (Steinheim, Germany). In all the experiments, nucleobases and nucleosides were dissolved in 1 N NaOH and added to the culture medium at the final concentration of 0.001 N NaOH whereas nucleotides were dissolved in aqueous solution. Geneticin was from Gibco (Invitrogen, Thermo Fisher Scientific, Milan, Italy); Deoxyribonuclease I amplification Grade (DNase, 1U/ul) was from Invitrogen (Thermo Fisher Scientific, Milan, Italy). Forodesine was purchased from D.B.A (Segrate, Italy). Tritiated GUA, [8-^3^H]-GUA (100 μCi/ml; specific activity >5 Ci/mmol), was purchased from Moravek Inc. (Brea, CA, United States).

### Cell cultures

The human tumoral cell lines were obtained from the American Type Culture Collection (ATCC, Teddington, UK). Human glioma cell lines, (U87-MG ATCC number: HTB-14 and U373-MG ATCC number: HBT-17) were cultured in RPMI 1640 (Cat. No. 61870-010, Gibco, Invitrogen); human amelanotic melanoma (C32, ATCC number: CRL 1585 and C32TG ATCC number: CRL 1579) in MEM (Cat. No. 41090-028, Gibco, Invitrogen) supplemented with non-essential amino acids (0.1 mM) (Cat. No. 11140-035, Gibco, Invitrogen) and sodium pyruvate (1 mM); C32TG cells were grown in medium supplemented with thioguanine (30 μM). Each growing medium was integrated with 10% (vol/vol) heat-inactivated foetal bovine serum (FBS, Cat. No. 10270-106, Gibco, Invitrogen) and penicillin-streptomycin (50 units-50 μg/ml). Due to serum supplementation, a final concentration of 0.5 μM GUA and 0.1 μM GUO was present in each growing media, evaluated by HPLC method as previously described (Giuliani et al., 2016); however, these concentrations can be considered negligible for experiments herein reported. The cell cultures were incubated at 37°C in a humidified 5% CO_2_ incubator and the culture medium was changed twice a week.

### BLAST search for purinoreceptor homology

Analysis of the GenBank^TM^ database containing the human genome sequence was performed by BLAST (Basic Local Alignment Search Tool, NCBI) program to search for orphan GPCRs sharing protein sequence similarity to human A_1_R, A_2A_R, A_3_R and P2Y_1_R were used as queries. Only orphan GPCRs (i.e., included in the IUPHAR list of class A orphan receptors at 2022) or de-orphanized GPCRs with evidence of possible multiple unknown endogenous ligands, showing protein sequence similarity to one or more purinoreceptors with an Expected (E) value <0.05 were selected from the BLAST list search. The list was further refined excluding those receptors not showing an expression in normal brain tissue and glioblastoma samples by analysis of RNA-sequencing data provided by The Cancer Genome Atlas (TCGA) (https://www.cancer.gov/about-nci/organization/ccg/research/structural-genomics/tcga). The mRNA expression of the selected receptors was further analyzed in glioma cell lines (i.e., U87 and U373) by qualitative RT-PCR.

### Tumor cell line RNAs and cDNA synthesis

Total RNA from U87 and U373 cell lines was extracted as previously described (Chomczynski and Sacchi, 1987). Total RNA from C32TG was extracted with the RNeasy plus mini kit (Qiagen, Hilden, Germany). Total RNA was treated with Deoxyribonuclease I amplification Grade to remove genomic contamination. Reverse transcription was performed using total RNA, RNase H-reverse transcriptase (Superscript II, Gibco BRL, Life Technologies, Gaithersburg, MD, United States) and random primer hexamers.

### Identification of GPCR mRNA expression by polymerase chain reaction (qualitative PCR)

First strand cDNA was subjected to PCR amplification using a set of specific primers corresponding to the nucleotide sequences of the GPCRs reported in the Supplementary Table S1.

The PCR program used was the following: a first denaturation step at 95°C for 5 min, followed by 35 cycles at 95°C for 1 min, 54–60°C (depending on annealing temperature of the primer set) for 2 min and 72°C for 3 min. Then, a final elongation step at 72°C for 10 min was performed. PCR products were examined by electrophoresis in 1.8% agarose 1x TAE gels and ethidium bromide or sybr-safe staining (Invitrogen).

### Quantitative real-time RT-PCR

Quantitative Real-time PCR (qRT-PCR) experiments were performed in the ABI Prism 7700 Sequence Detection System from Applied Biosystems. For each GPCR mRNA expression, three sequence-specific oligonucleotides were designed using the Primer Express oligo design software (Applied Biosystem, Carslbad, CA, United States). Two of them were forward (Fw) and Reverse (Rv) primers used for PCR amplification. The third sequence (TaqMan Probe, Applied Biosystem) was a fluorogenic probe labelled with a fluorescent reporter dye (6-FAM) and a quencher dye (TAMRA) attached at the 5′and 3′ end, respectively. The probe was designed to hybridize the portion of PCR amplicon between Fw and Rv primers. The primers and probe used are reported in Supplementary Table S2.

The difference in the initial amount of total RNA between the samples was normalized in every assay using glyceraldehydes-3-phosphate dehydrogenase housekeeping gene expression as an internal standard (TaqMan Human GAPDH Control Reagent, Applied Biosystem). Each PCR reaction was carried out in 50 μl final volume using TaqMan Universal PCR Master Mix (Applied Biosystems), 900 nM of primers and 200 nM of probe. Finally, 2.5 μl of diluted cDNA (1/10 vol/vol) was added for each reaction. Each sample was loaded in triplicate. Then, standard conditions were used for PCR amplification (50°C for 2 min, 95°C for 10 min, followed by 50 cycles at 95°C for 15 s, 60°C for 1 min). Reactions without cDNA were performed as negative control (No Template Control, NTC). Glyceraldehyde-3-phosphate dehydrogenase (GAPDH) PCR amplification was carried out under the same conditions, except that for concentrations of primers and probe, in which 100 and 200 nM were used, respectively. Relative quantification of RNA expression was calculated using comparative C_T_ method based on the threshold cycles (Ct values) of the gene of interest and of the internal reference gene (GAPDH).

### siRNAs design and synthesis

siRNAs corresponding to GPR3, GPR21, GPR22, GPR23 and LPAR6/P2Y_5_ mRNAs as well as those corresponding to hypoxanthine-guanine phosphoribosyl transferase (HGPRT) (GenBank accession number NG_012329.1) gene were designed according to the report of Elbashir et al. (2001). The siRNAs used for GPCRs and HGPRT are listed in Supplementary Table S3. RNA not complementary to any cellular transcript (ctrl RNA) was used as control (see Supplementary Table S3). The sequences were subjected to BLAST search to confirm the absence of homology to other additional known coding sequences in the genome human project. siRNAs were chemically synthesized by MWG Biotech AG (Ebersberg, Germany) and resuspended according to the manufacturer’s instructions.

### siRNA transfection, treatments and MTT colorimetric assay

Glioma cells were transfected with siRNAs at 50% confluency using Oligofectamine (Invitrogen). The day before transfection, the cells were trypsinized, counted, plated at 4 × 10^3^ cells/well in 96 well plates “Nunclon TM Microwell TM” (Nunc, Roskilde, Denmark) in the medium containing 10% FBS, and were incubated at 37°C. After 24 h, cells were transfected according to the manufacturer’s procedure in the absence (mock transfected), in the presence of the ctrl RNA, or in the presence of specific siRNAs (final concentration 50 nM). After 24 h, the transfected cells were exposed to GBPs at the concentrations indicated in the related figures. Microplates were incubated at 37°C in a humidified 5% CO_2_ incubator for 24, 48 and 72 h and then the growth inhibition was measured with a colorimetric assay based on the use of tetrazolium salt MTT (3-(4,5-dimethylthiazol-2-yl)-2,5-diphenyl tetrazolium bromide) (Mosman, 1983), currently used also to measure drug-induced cytotoxicity. The results were read on a multiwell scanning spectrophotometer ((Spectracount^TM^, PerkinElmer, Waltham, MS, United States), using a wavelength of 570 nm. Each value was the average of 4-10 wells (standard deviations were less than 20%). The % of cell growth was calculated according to NCI (National Cancer Institute, Bethesda, Maryland): 100 x (T-T_0_)/(C-T_0_) (T is the optical density of the test well after an established period of exposure to test compound; T_0_ is the optical density at the beginning of the treatment with GUA, GUO, GMP; C is the optical density of the controls). IC_50_ was calculated using GraphPad Prism version 6.0 for Windows, after fitting the dose-response data to a sigmoidal curve (nonlinear regression).

### Human GPR23: Subcloning in expression vectors and generation of stably transfected cell lines

The PCR product containing the entire coding sequence of the human GPR23 mRNA was cloned into the expression vector pcDNA^TM^ 3.1D/V5-His-TOPO (Invitrogen, Scotland, UK). The GPR23 ORF fragment was amplified with the following primers: Fw 5′-CAC​CAT​GGG​TGA​CAG​AAG​AT-3′ and Rv 5′-TGCTAGAATC CACCTTTTAG-3’. To confirm the orientation of the GPR23 insert, the construct, pcDNA 3.1 D/V5-His-TOPO/GPR23 was subjected to restriction enzyme digestion in the appropriate analysis buffer with BamH I (Promega, Madison, WI, United States) and, to double digestion with XbaI/HindIII (Promega). The digestion products were analyzed by electrophoresis in 0.8% agarose 1 x TAE gel and ethidium bromide staining. Moreover, to confirm the identity of the GPR23 entire open reading frame, DNA sequencing was performed by standard fluorescent dideoxy chain-termination procedure with the Abi Prism 377 automatic sequencer (Thermo Fisher). MACAW alignment allowed us to compare the sequence of the insert with the GPR23 gene. U87 and U373 cell lines were stably transfected with the pcDNA 3.1D/V5-His-TOPO/GPR23, or with the control vector, pcDNA 3.1D/V5-His-TOPO/*lac*Z, using lipofectamine reagent (Invitrogen) according to the manufacturer’s procedure. C32TG cell lines were stably transfected with the pcDNA 3.1 D/V5-His-TOPO/GPR23 or with the control vector, pcDNA 3.1D/V5-His-TOPO/*lac*Z, using oligofectamine reagent (Invitrogen). 48 h after transfection, cells were cultured in the growth medium supplemented with geneticin (G418, 600 μg/ml). After 1–2 weeks, individual clones were isolated to select clones with the highest expression of receptors. The different clones were maintained in their medium containing 500 μg/ml G418. GPR23 mRNA expression was evaluated by qualitative PCR and qRT-PCR, as previously described (Yin et al., 2009; Garozzo et al., 2010). For GUA dose-response experiments, two different overexpressing stable clones were used for U87 (U87cl12, U87cl8), U373 (U373cl6, U373cl18), and C32TG cells (C32TGcl10, C32TGcl19). The parental cell lines (wtU87-wtU373-wtC32TG) and clones derived by stable transfection of the lacZ gene (U87-, U373-, C32TG-lacZ) were used as controls.

### Detecting of GPR23-tagged V5 protein by immunofluorescence

To allow the expression of native protein in GPR23-expressing stable U87 cell line (U87-GPR23), we used the Tag-On-Demand System (Invitrogen). One day before transduction, 10^4^ U87-GPR23 cells were plated in chamber slides (Nunc, Roskilde, Denmark) in 100 μl of complete growth medium and incubated at 37°C overnight. On the day of transduction, the growth medium was removed from each well of cells and replaced with 50 μl of fresh growth medium. Then the adenovirus was added to each well using a Multiplicity of Infection (MOI) of 50 and cells were incubated for 5–6 h at 37°C. Afterwards, the medium containing virus was removed from the cells and fresh complete growth medium was added to the cells.

Assay for C-terminally-tagged recombinant protein expression was performed 24 h post-transduction, with immunofluorescence, using Anti-V5-FITC conjugated antibodies (Invitrogen) prepared by crosslinking the appropriate primary antibody with the FITC fluorophore. Briefly, cells were washed twice with PBS and fixed by adding 200 μl of room temperature100% methanol. After incubation, cells were washed 5 × 2 min with PBS and, to reduce non-specific binding of antibody, were incubated for 20 min at room temperature with 200 μl of blocking solution (PBS containing 10% FBS). Then, 100 μl of blocking solution containing the 1:2000 dilution of antibody were added and the mixture was incubated for 1 h at room temperature in the dark. Cells were washed again 2 × 5 min with PBS and observed cells with a fluorescence microscope (Zeiss Axio Scope A1, White Plains, NY, United States) equipped with a FITC filter.

### [^3^H] guanine binding assay

We started with the preparation of membranes from U87 cell lines, according to the previously published procedure (Traversa et al., 2002; Frinchi et al., 2020). Cultured cells (1 × 10^6^ cells) were collected and homogenized in ice-cold buffer (0.25 M sucrose, 5 mM HEPES pH 7.4) by teflon-glass homogenizer (350 rpm, 7 up and down strokes). The homogenate was centrifuged at 1000×*g* (10 min, 4°C). The pellet, resuspended in 2 ml buffer, was centrifuged as aforementioned and the supernatants were pooled and centrifuged at 11,000×*g* (20 min, 4°C). The pellet was resuspended in cold HEPES (20 mM, pH 7.4) and centrifuged at 100,000×*g* (30 min, 4°C). The final pellet, resuspended in the same buffer to a final protein concentration of 100 μg per 1 ml, was aliquoted (100 μl per vial) and stored at −80°C. Before using in binding experiments, the membranes were washed twice with PBS buffer (8.8 mM, pH 7.4). The protein concentration was determined by the Bradford method (Bradford, 1976). Subsequently, the measurement of [^3^H]-GUA binding was carried out. Membranes (50 μg) were preincubated (10 min, 25°C) in binding buffer (20 mM Tris-HCl, 1 mM EGTA, 5 mM MgCl_2_, 100 mM NaCl, pH 7.4) and then incubated (30 min, 25°C) with [^3^H]GUA in a total volume of 0.5 ml binding buffer. For saturation experiments, a concentration range of 6.25–300 nM [^3^H]-GUA was used. Nonspecific binding was determined by adding 500 μM unlabeled GUA and specific binding was calculated by subtracting nonspecific from total binding. In competition experiments, displacing agents at different concentrations and 50 nM [^3^H]-GUA were added, and the reaction started by adding the membranes. After 30 min, the reaction was stopped by adding 3 ml of cold binding buffer. The samples were rapidly filtered by vacuum filtration using Whatman GF/B glass fiber filters. These filters were then washed four times (2.5 ml cold binding buffer each time), dried for 1 h at 30°C, transferred in scintillation vials filled with 5 ml of Filter count scintillation cocktail (Beckman Coulter, Inc.; Fullerton, CA, United States). Bound radioactivity was measured in a Beckman Coulter LS6500 Multipurpose Scintillation Counter (Beckman Coulter, Inc.). For saturation and displacement curves, the pooled data were fitted by a computerized nonlinear regression analysis and resolved for the presence of a single high-affinity binding site.

### Enzyme assay to determine PNP activity in the extracellular medium of U87 glioma cells

Samples containing PNP were obtained as previously described by Giuliani et al. (2016). Briefly, U87 glioma cells were incubated in serum-free medium. After 1, 6 and 12 h, an aliquot of medium was taken and the enzyme present was concentrated using Amicon Ultra 2 ml filters (cutoff 10 K, Merck Life Science, Milan, Italy), while cells were scraped in lysis buffer (5 mM HEPES pH 8.5, 2 mM EDTA, and protease inhibitor cocktail) and sonicated to obtain cytosolic extracts. Protein content was quantified using a colorimetric protein assay kit (Bio-Rad, Segrate, Italy). PNP activity was evaluated by measuring the transformation of GUO, the enzyme’s substrate, into GUA by HPLC analysis. The enzymatic reaction was performed in HEPES (50 mM; pH 7.0) containing 50 mM inorganic phosphate plus an aliquot of the concentrated medium as a source of PNP. 100 µM GUO was then added and the mixture was incubated by shaking at 37°C for 15 min. The reaction was stopped by heating the mixture at 70°C for 5 min. After centrifugation, the supernatant was filtrated before HPLC analysis. The HPLC (Agilent 1100 Series, Waldbronn, Germany) was equipped with a thermostated column compartment, a diode array detector, and a fluorescence detector. The separation was achieved by a Phenomenex Kinetex pentafluorophenyl analytical column (Phenomenex INC.; Bologna, Italy) kept at 35°C and applying a 15-min nonlinear gradient with a flow rate of 1 ml/min (for further details see Giuliani et al., 2017). The excitation and emission wavelengths for monitoring fluorescent GUO and GUA were 260 and 375 nm, respectively. PNP activity is usually defined as international units (IU), 1 IU corresponding to the amount of the enzyme that catalyzes the conversion of 1 μmol of substrate per minute under the specified conditions of the assay method. Accordingly, given the small amount of cells and thereby of PNP activity, we expressed it as micro-international units (µIU), 1 µIU corresponding to the amount of PNP that catalyzes the conversion of 1 pmol of substrate per min (pmol/min).

### Statistical analysis

All experiments were usually performed from three to four times, unless differently specified. The quantitative data are expressed as mean ± SD. Time-response curves were calculated by using nonlinear regression (GraphPad Prism 6.0 software, San Diego, CA, United States). Statistical analyses were performed by Prism 6.0 software, using Student’s t test coupled to the Holm-Sidak method or two-way analysis of variance (ANOVA). Group differences with *p* < 0.05 were considered statistically significant.

## Results

### BLAST search for human class a orphan GPCRs with purinoreceptor similarity

Since GBP signalling has been somewhat related to certain purinergic receptors, we aimed to identify human class A GPCRs with only preliminary evidence of the existence of an endogenous ligand or as-yet unidentified, showing sequence similarity to purinoreceptors. To this end, a bioinformatic search was performed by BLAST, using as a query the protein sequences of human A_1_R, A_2A_R and A_3_R responsive to ADO and P2Y_1_R responsive mainly to ADP (Supplementary Data S1). Thirty-three GPCRs showing similarity to one or more ADO or P2Y_1_ receptors, with an E value <0.05 (matrix BLOSUM 45), were selected from the BLAST search list (Supplementary Data S2) according to multiple criteria: 1) they belonged to the class A orphan receptors according to IUPHAR/BPS Guide to PHARMACOLOGY (http://www.guidetopharmacology.org/GRAC/FamilyDisplayForward?familyId=694), or 2) they belonged to a group of de-orphanized GPCRs (LPAR4,5,6) for which experimental evidence of additional unknown endogenous ligands was available (Noguchi et al., 2003; Lee et al., 2007; Yanagida et al., 2007) and 3) they were expressed in normal brain tissues and in glioblastoma samples (averaged transcript per kilobase million (TPM) >0.1 in glioblastoma samples and normal brain tissues of the TCGA consortium). Subsequently, the mRNA expression of the selected orphan GPCRs was analysed by qRT- PCR in human glioma cell lines (U87, U373), which were previously reported to be responsive to GUO (Garozzo et al., 2010). Four receptors, namely GPR21, GPR22, GPR23 and LPAR6/P2Y_5_R, expressed at relatively high levels in both cell lines, were then selected for further experiments. In addition, a GPCRs expressed only in U87 cells, namely GPR3, was also included.

### Silencing selected GPCRs in U87 cell line

Subsequently, the mRNA expression of the selected receptors, namely GPR3, GPR21, GPR22, GPR23 and LPAR6/P2RY5, was silenced in U87 cell line by short interference RNA (siRNA) methodology (25). To this end, a pair of specific siRNAs targeting the coding region of each receptor was designed and synthesized (Supplementary Table S3). Thus, as shown in Supplementary Figure S1, GPR3, GPR21, GPR22, GPR23 and LPAR6/P2Y_5_ mRNA levels, measured 48 h after siRNA lipo-transfection into the U87 cell line, were persistently decreased by about 75%, 72%, 90%, 75% and 90%, respectively. Importantly, the downregulation of the selected receptors did not affect cell survival, except for a significant cell growth inhibition (38%) observed with GPR21 silencing (data not shown). Overall, these results validated our experimental strategy for silencing orphan GPCRs in the U87 cell line.

### Effect of silencing orphan GPCRs in GBP-mediated growth inhibition of U87 cells

**FIGURE 1:**
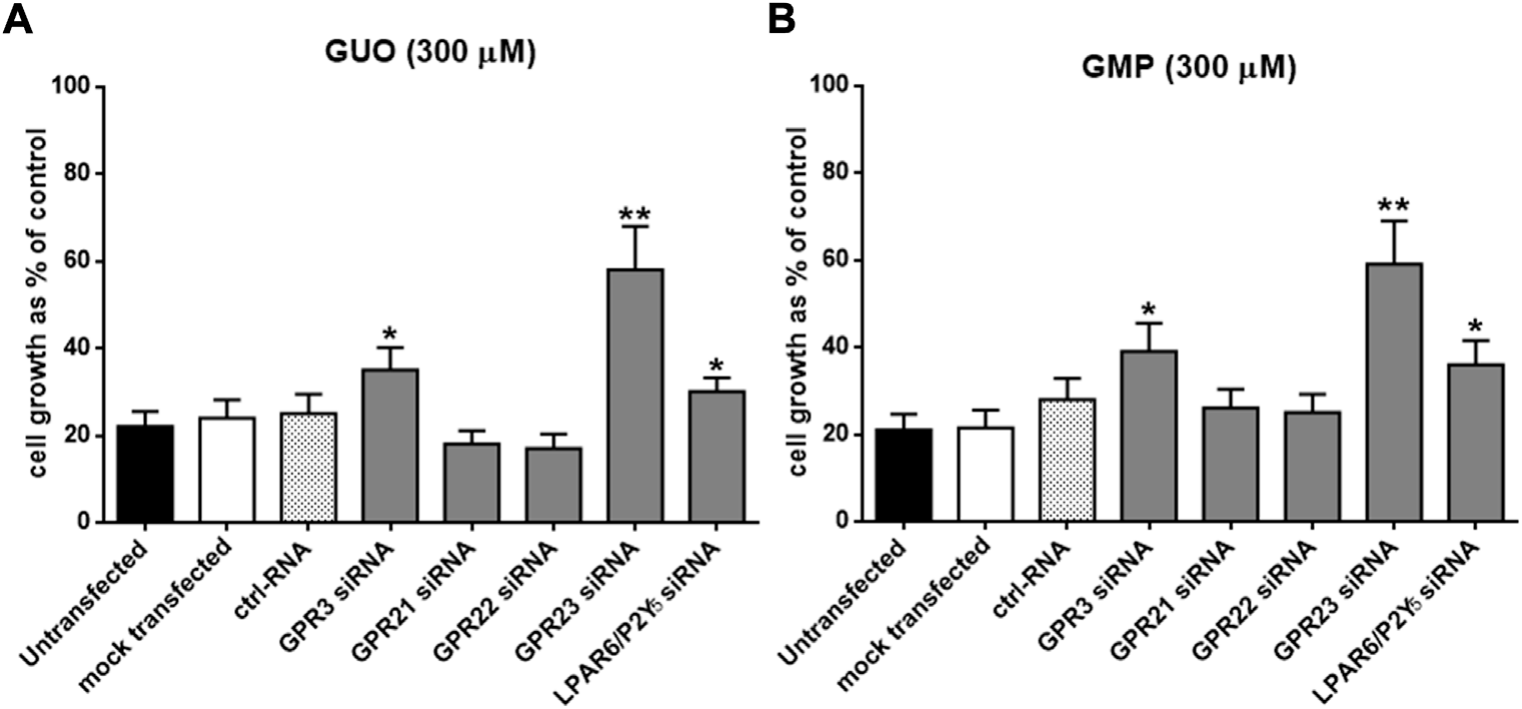
Effects of lipotransfection with different GPCR-siRNAs on sensitivity to GUO **(A)** and GMP **(B)**. U87 cells, grown up to 50% confluency into 96 multiwell plates, were lipotransfected in the absence of siRNAs (mock transfected), in the presence of 50 nM ctrlRNA or in the presence of 50 nM GPR3-siRNA, GPR21-siRNA, GPR22-siRNA, LPAR6/P2Y_5_-siRNA and GPR23-siRNA and then incubated in growth medium. After 24 h cells were treated with GUO or GMP (300 μM) for 72 h. Cell growth was evaluated by the MTT assay. Results are expressed as the percentage of cell growth evaluated in untreated cells (control). Each point represents the mean ± SD of 3 independent experiments. Statistical significance: *p < 0.05 **p < 0.01 vs. mock transfected cells (Student’s t test).

A functional signature of GBPs, as compared to other purines, is the profound antiproliferative effect on human tumoral cell lines, including U87 cells (Garozzo et al., 2010). Accordingly, we questioned if silencing the selected orphan GPCRs would have any impact on the GBP-mediated growth inhibition of U87 cells. Thus, twenty-four hours after siRNA transfection, U87 cells were exposed to GUO and GMP (300 µM) for 24, 48, or 72 h and, at the end of the treatment, cell proliferation was assessed through the MTT assay. Indeed, GUO and GMP treatment triggered a maximal antiproliferative effect of ∼80% after 24 h which lasted until 72 h upon incubation (Figure 1), in agreement with previous data (Garozzo et al., 2010). The inhibition of cell proliferation in non-transfected cells was similar to that observed in mock and ctrl-RNA transfected cells, ranging from 70 to 80%. Importantly, the antiproliferative effect of GUO and GMP after 72 h of treatment was significantly reduced (∼50%; *p* < 0.01) in cells silenced for GPR23 (Figures 1A,B **)**. In contrast, silencing of GPR21 or GPR22 receptors did not precluded GUO or GMP antiproliferative effects, while that of GPR3 and LPAR6/P2Y_5_ receptors, although significant (*p* < 0.05), had a limited effect on GUO- and GMP-mediated cell growth inhibition (5%–15% reduction). Collectively, these results suggested that GPR23 might play a potential role in GBP-mediated antiproliferative effects of U87 cells, thus we subsequently focus on this receptor.

### Pharmacological characterization of GPR23-dependent antiproliferative effect in U87 cells

As shown previously, silencing of the GPR23 had an impact in the GBP-mediated growth inhibition of U87 cells. Now, we aimed at pharmacologically characterizing this GPR23-dependent antiproliferative effect by constructing concentration-response curves for GUO, GMP and GUA. To this end, U87 cells were GPR23 silenced or mock transfected before being exposed to increasing concentrations of GUO, GMP and GUA for 72 h and the cell proliferation assayed. Interestingly, we also included GUA as this compound, which usually derives from the metabolism of GUO, has been shown to be an active GBP in other cell lines (Wengert et al., 2011; Giuliani et al., 2012).

**FIGURE 2:**
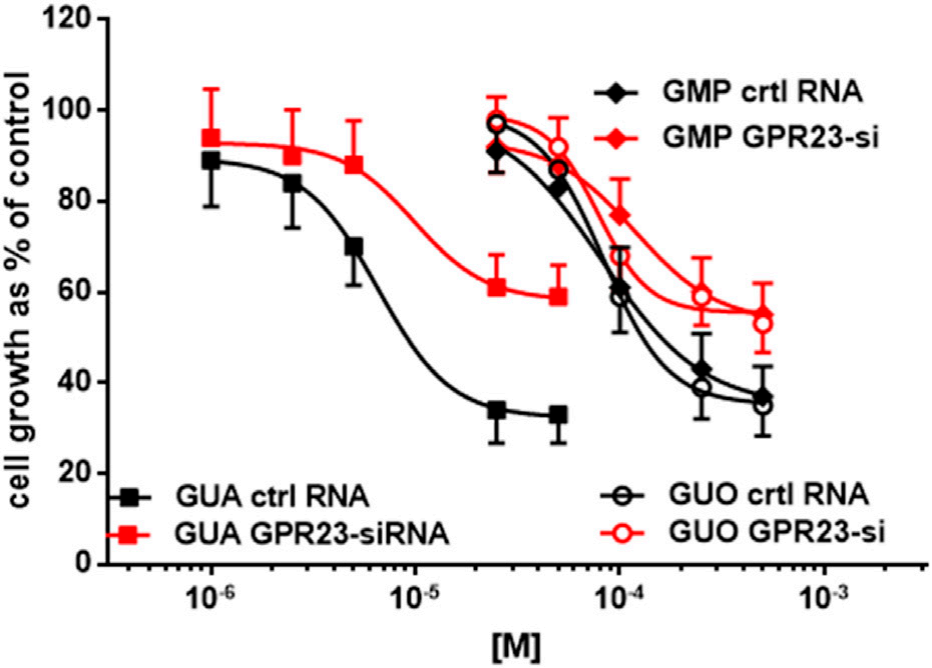
Effect of lipotransfection with GPR23-siRNA on sensitivity to GUA, GUO and GMP. Twenty-four hours after lipotransfection with GPR23-siRNA, U87 cells were treated with GUO or GMP (50–500 μM) or GUA (1–50 μM) for 72 h. Results are expressed as the percentage of cell growth evaluated in untreated cells (control) by the MTT assay. Each value is the mean ± SD of 10 different samples. Statistical significance of data obtained in GPR23-siRNA cells vs. control (ctrl) RNA transfected cells: *p* < 0.001 for data relating to GUA effect and *p* < 0.01 for those relating to GUO and GMP effects (two-way ANOVA test).

All three GBPs caused a concentration-dependent inhibition of proliferation in mock transfected U87 cells and with similar efficacy (i.e., maximal growth inhibition of 80%) (Figure 2). Indeed, the antiproliferative effect induced by GUA, GUO, or GMP was concentration-dependent also in GPR23-silenced cells, but significantly lower than that observed in the respective controls, as expected. Remarkably, GUA showed to be the most potent GBP inducing growth inhibition both in control (i.e., transfected with RNA not complementary to any cellular transcript, ctrlRNA) and GPR23 silenced U87 cells. Accordingly, IC_50_ values for GUA, GUO, or GMP were 40 μM, ∼ 190 and ∼170 μM, respectively. Overall, these results indicated that GPR23 silencing modified the antiproliferative efficacy of GBPs but not their potency (Figure 2), thus pointing to GUA as the most reliable ligand for GPR23.

### Evaluation of GBPs-mediated antiproliferative effects upon heterologous expression of GPR23 in U87 and U373 cells

**FIGURE 3:**
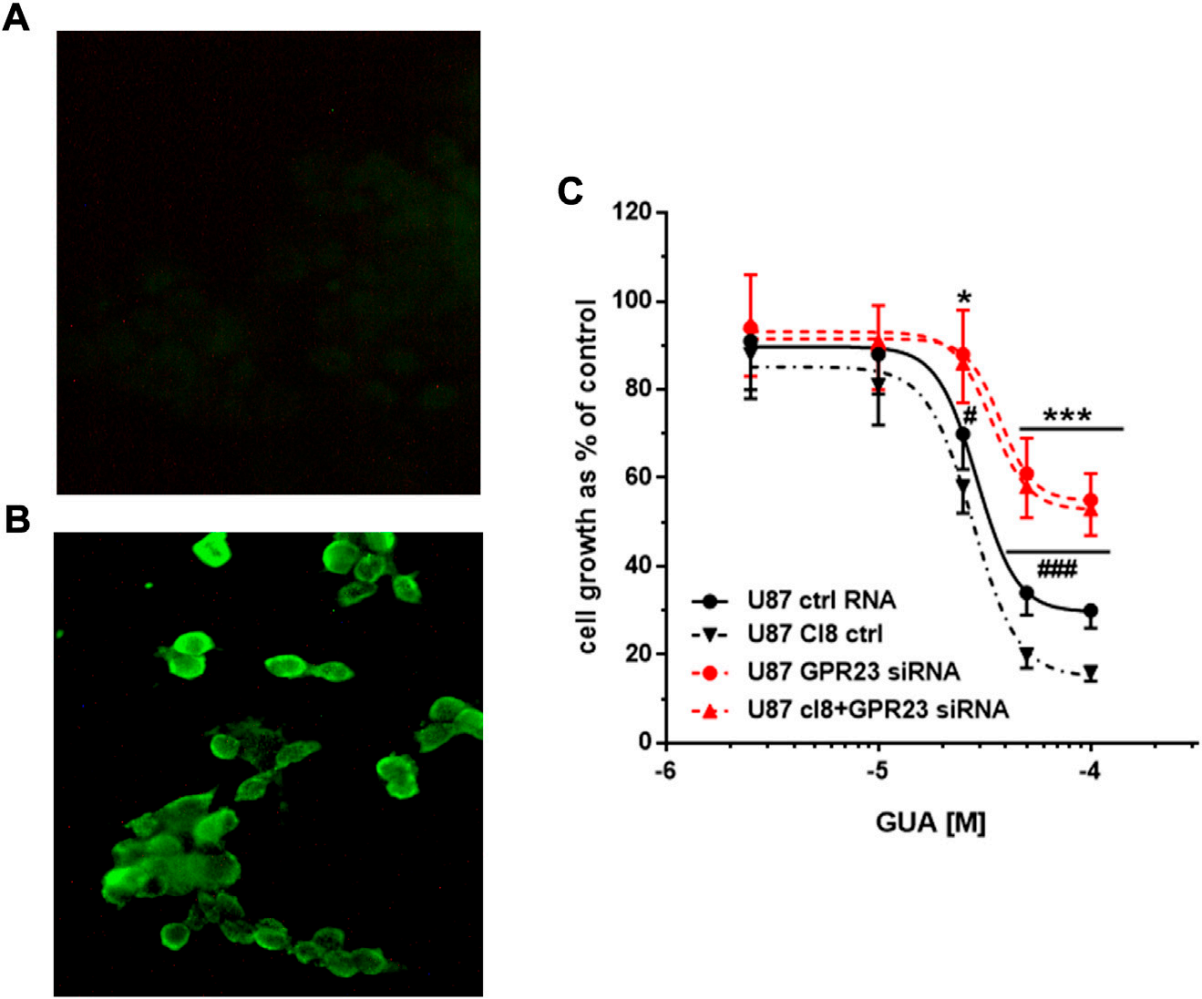
Effect of GPR23 overexpression on sensitivity to GUA. **(A,B)** Immunofluorescence microphotographs of a GPR23-overexpressing U87 cell clone (U87cl12). Expression of V5-tagged recombinant GPR23 was induced in U87cl12 cells by TAG on Demand adenoviral transduction and detected by the anti-V5-FITC conjugated antibody. **(A)** not-transduced U87cl12 cells stained with anti-V5-FITC antibody; **(B)** transduced U87cl12 cells stained with anti-V5- FITC antibody. The images are representative of six independent experiments performed with different clones overexpressing GPR23, which gave similar results. **(C)** Following stable overexpression of GPR23, some of these cells (U87cl8) were lipotransfected in the presence of 50 nM ctrlRNA (ctrl RNA) or GPR3-siRNA (GPR23siRNA). Twenty-four hours after this procedure, all cells were exposed to GUA (1–100 μM) for 72 h. Results, expressed as the percentage of cell growth evaluated in untreated cells (control) by the MTT assay, are the mean ± SD of 4 different experiments for each cell type. *p < 0.05 **p < 0.001 and ^#^ *p* < 0.05 ^###^ *p* < 0.001: statistical significance vs. U87 ctrl RNA and U87 cl8, respectively (Student’s t test).

Conversely, to support the dependence of the GUA-induced inhibition of cell growth on GPR23 activation we assessed GUA effects in U87 and U373 cells overexpressing this receptor. To this end, U87 or U373 wild type (wt) cells were transfected with lacZ control vector or with the cDNA encoding the GPR23 receptor (U87 clones 12 and 8; U373 clones 6 and 18). GPR23 protein density was monitored upon receptor transduction in U87 and U373 cells by immunofluorescence detection. This assay showed, for instance, a superior expression of V5-tagged GPR23 receptor in U87 cells clone 12 (U87cl12) (Figure 3B). Similar results were obtained with other clones such as U87cl8, U373cl6 and U373cl18 cells. Thus, these clones showing the highest GPR23 mRNA expression were used for the next experiments. As controls, the parental cell lines (wtU87 and wtU373) and clones derived by stable transfection of the lacZ gene (U87lacZ and U373lacZ) were used (Figure 3A).

Subsequently, the GUA-mediated antiproliferative effect in GPR23 overexpressing U87 and U373 cells was determined by the MTT assay (Table 1). As expected, while no significant difference was found among the IC_50_ values related to the GUA effect in any cell line assayed, a significant increment in the GUA-induced growth inhibition was observed in U87cl12, U87cl8 and U373cl18 cells (Table 1).

**TABLE 1:** Inhibitory growth effect caused by GUA in U87 or U373 cell lines, wild type (WT) or overexpressing GPR23 receptor (U87 clones 12 and 8; U373 clones 6 and 18) or lacZ control vector.

Drug under evaluation		U87 cells				U373 cells		
GUA	WT	LacZ	cl12	cl8	WT	lacZ	cl6	cl18
IC_50 (_µM)	37.1 ± 2.25	39.4 ± 9.11	32.7 ± 4.48	30.3 ± 3.73	49.5 ± 7.42	56.9 ± 5.55	49.5 ± 3.46	43.5 ± 4.43
Efficacy at the IC_50_ concentration	61.2 ± 4.02	57.4 ± 6.21 (NS)	75.1 ± 3.13 (p < 0.05)	82.5 ± 4.47 (p < 0.01)	57.5 ± 4.34	56.3 ± 7.71 (NS)	60.6 ± 5.33 (NS)	77.4 ± 8.12 (p < 0.05)

Cells were treated with increasing concentrations of GUA (1–500 µM) for 72 h before the proliferation inhibition was determined by MTT assay. Accordingly, IC_50_ values were calculated for each cell type and reported in the Table. Subsequently, the effect of GUA administered at its IC_50_ for 72 h on the growth of each cell type was determined by MTT assay and expressed as the percentage of cell growth in untreated cells (used as control). All values are the mean± SD of n. 3 independent experiments. Statistical significance evaluated by Student’s t test (*p* values are indicated between brackets; NS, not significant difference).

Importantly, in U87cl8 cells transfected with GPR23-siRNA the GUA antiproliferative efficacy was dramatically decreased as compared to the effect obtained in U87 transfected with control RNA and the obtained concentration-response curve was very similar to that of wt-U87 cells transfected with GPR23 siRNA (Figure 3C). Similar results were obtained for U373cl18 cells. Overall, these findings provided complementary evidence indicating the involvement of GPR23 in GBPs-mediated antiproliferative effects.

### Outlining the role of GUA in GPR23-mediated effects

**FIGURE 4:**
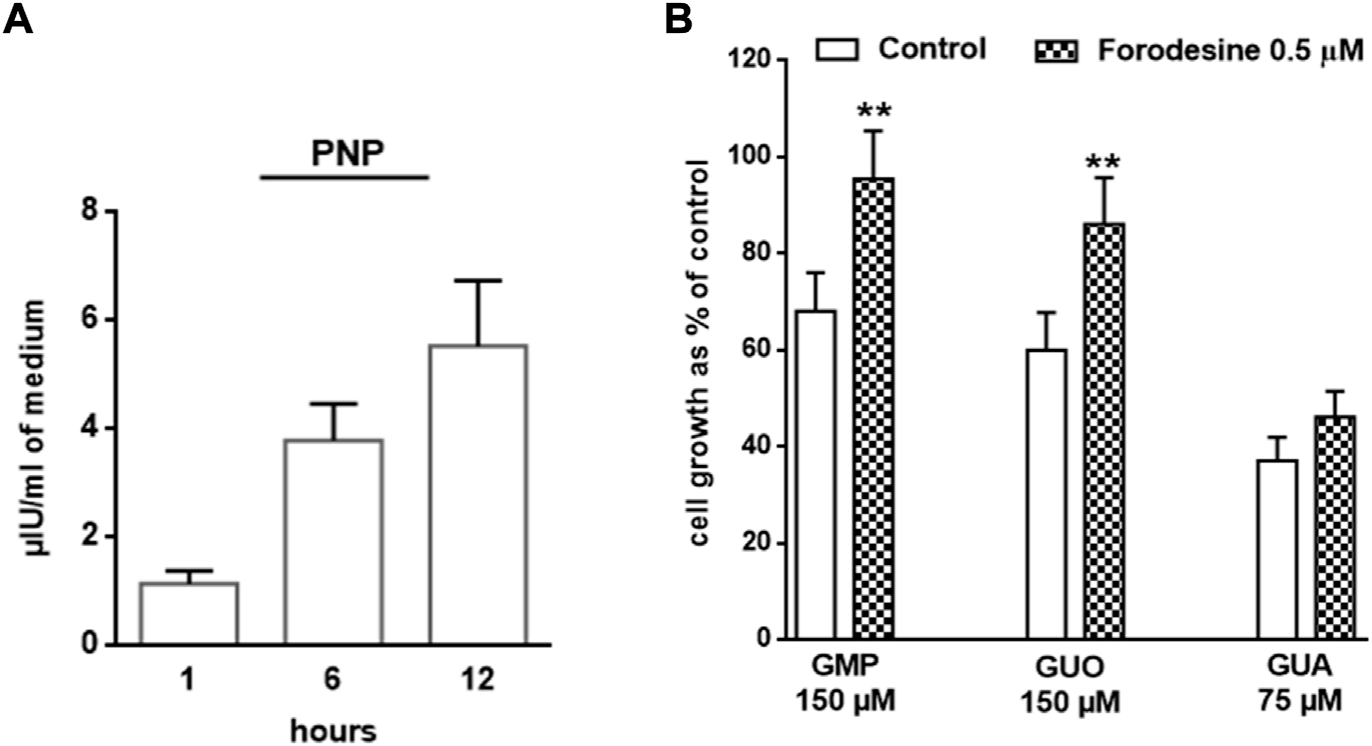
Involvement of PNP activity in GBPs-mediated U87 cell growth inhibition. **(A)** Activity of PNP present in the U87 cell culture medium. The growth medium of cells was collected at different times (i.e., 1, 6 and 12 h) and the PNP activity determined (*see* Materials and Methods). **(B)** Impact of PNP inhibition on GBPs-dependent antiproliferative effect. U87 cells were exposed to GMP, GUO or GUA for 72 h, in the presence or absence (w/t) of forodesine, a PNP inhibitor. Results are expressed as the percentage of cell growth evaluated by the MTT assay in untreated cells (control). All values are the mean ± SD of 4 independent experiments. Statistical significance: **p < 0.01 vs. cells not treated with forodesine (Student’s t test).

To further define the prevalent GPR23-dependent GUA role on U87 cell growth, we questioned whether the effects of GUO and GMP were intertwined with those of GUA. It is well-known that GMP and GUO catabolism ends into GUA production by the activity of the PNP. This enzyme is secreted to the extracellular milieu by different neural cells (10, 26), thus we assessed whether U87cells also secrete PNP. Indeed, extracellular PNP activity increases in a time dependent fashion in the culture medium of U87 cells (Figure 4A).

Importantly, when U87cells were pre-treated with forodesine, a selective PNP inhibitor, the antiproliferative effects of GMP and GUO, but not that caused by GUA, were abolished (Figure 4B). These results suggested that GUA behaves as an endpoint effector of GBPs-mediated antiproliferative effects.

**FIGURE 5:**
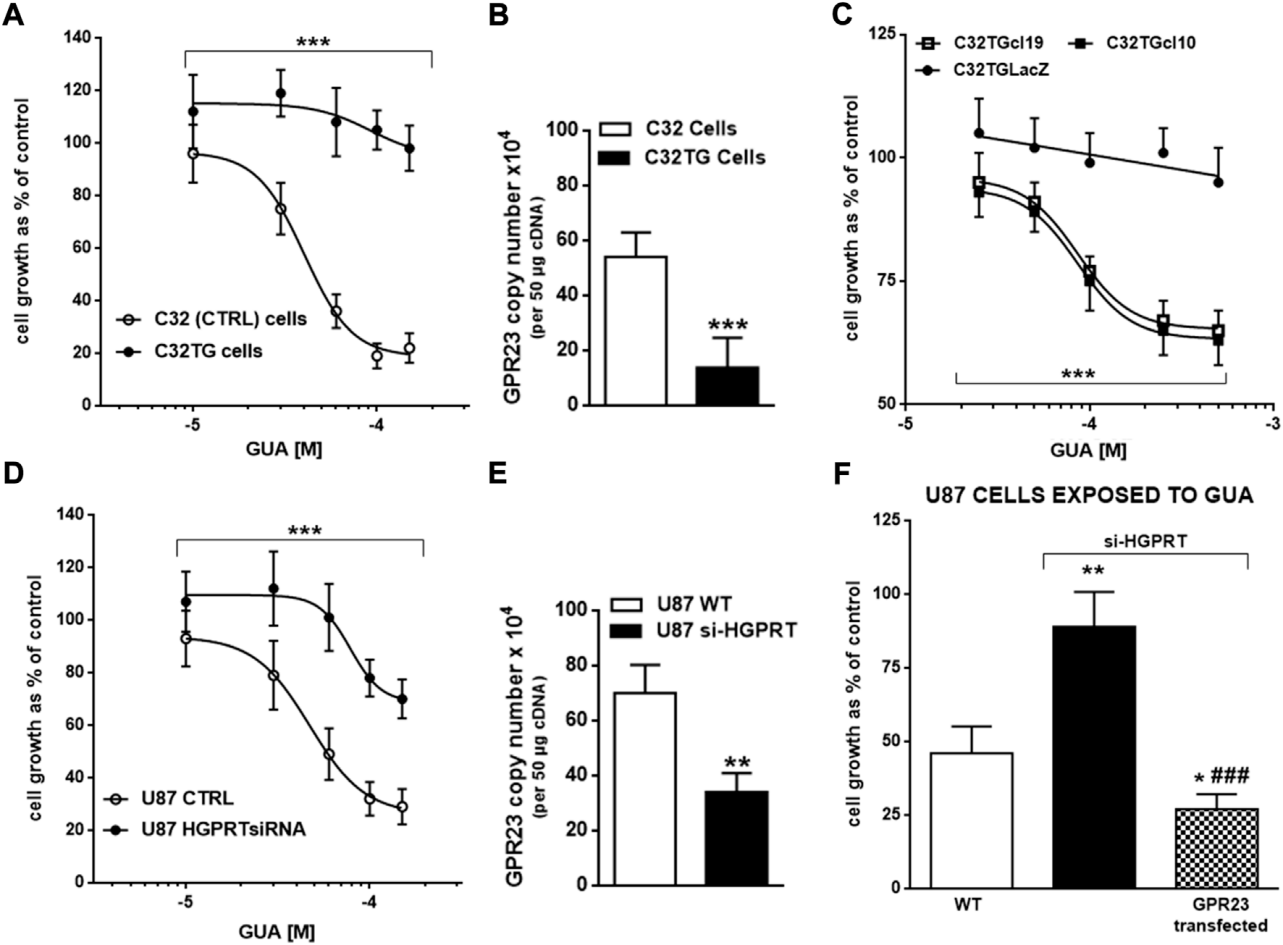
Involvement of HGPRT enzyme in GUA-mediated cell growth inhibition. Concentration-response curves of GUA-mediated antiproliferative effect in **(A)** C32 melanoma cells expressing normal (WT, wild type) or inactive HGPRT form (C32TG cells) or **(B)** U87 wild type cells (WT) or silenced for HGPRT (U87 HGPRTsiRNA). Furthermore, the mRNA expression of GPR23 was evaluated in C32 **(C)** and U87 **(D)** cells by qRT-PCR. Finally, **(E)** C32TG cells with mutated HGPRT were transfected to overexpress GPR23 (C32TGcl19 and C32TGcl10) or LacZ construct (C32TGLacZ). Likewise, **(F)** U87 WT or HGPRT silenced cells were transfected to overexpress GPR23. Afterwards, cells were exposed to GUA as described above. For the entire duration of experiments, C32TG, -LacZ cells and GPR23 overexpressing clones were cultured in the medium without thioguanine. Results are expressed as the percentage of cell growth evaluated by the MTT assay in untreated cells (control) in **(A,B,E,F)** Panels. In all panels, values represent the mean ± SD of 3 independent experiments. Statistical significance: ***p < 0.001 vs. C32 or U87 WT cells (as for Panels **(A,B)**) or vs. C32TGLacZ cells (only as for Panel **(E)** (ANOVA two-way test); **p < 0.01 and ***p < 0.001 vs. C32 cells with normal HGPRT form or U87 WT cells; ^###^ *p* < 0.001 vs. U87 si-HGRPT cells (Student’s t test as for panels **(C,D,F)**.

Since nucleobases are recovered by cells to be reused intracellularly, we evaluated the involvement of HGPRT, a key enzyme in the purine salvage pathway, in the GUA-mediated antiproliferative effect. Interestingly, HGPRT, by converting GUA and hypoxanthine into GMP and inosine monophosphate, respectively, regulates GUA levels. To this end, C32 melanoma cells bearing an active or inactive (C32TG cells) form of this enzyme were used to assess the impact of HGPRT activity on GUA-mediated inhibition of cell growth. Interestingly, while GUA inhibited concentration-dependently the proliferation of C32 cells, it was ineffective in C32TG cells (Figure 5A). Conversely, silencing HGPRT in U87 cells significantly reduced the antiproliferative efficacy of GUA (Figure 5B). Overall, these results suggested an involvement of this purine salvage enzyme in GUA signaling, in agreement with previous findings reported by Garozzo et al. (2010).

Next, to ascertain the relationship of HGPRT with GUA signaling we determined GPR23 mRNA expression in cells lacking HGPRT activity. Importantly, C32TG cells showed very low expression of GPR23 as compared to C32 cells (Figure 5C). In addition, silencing HGPRT in U87 cells significantly reduced the mRNA expression of GPR23 (Figure 5D). Conversely, GPR23 transfection of C32TG and U87 cells silenced for HGPRT expression rescued the GUA-mediated antiproliferative effect (Figures 5E,F). These results may suggest that HGPRT regulation of GUA levels will define GPR23 content and signaling. Collectively, these findings reinforce the idea that GUA effect on cell growth would mediate by GUA interaction with GPR23.

### GUA binding to U87 cell membranes

**FIGURE 6:**
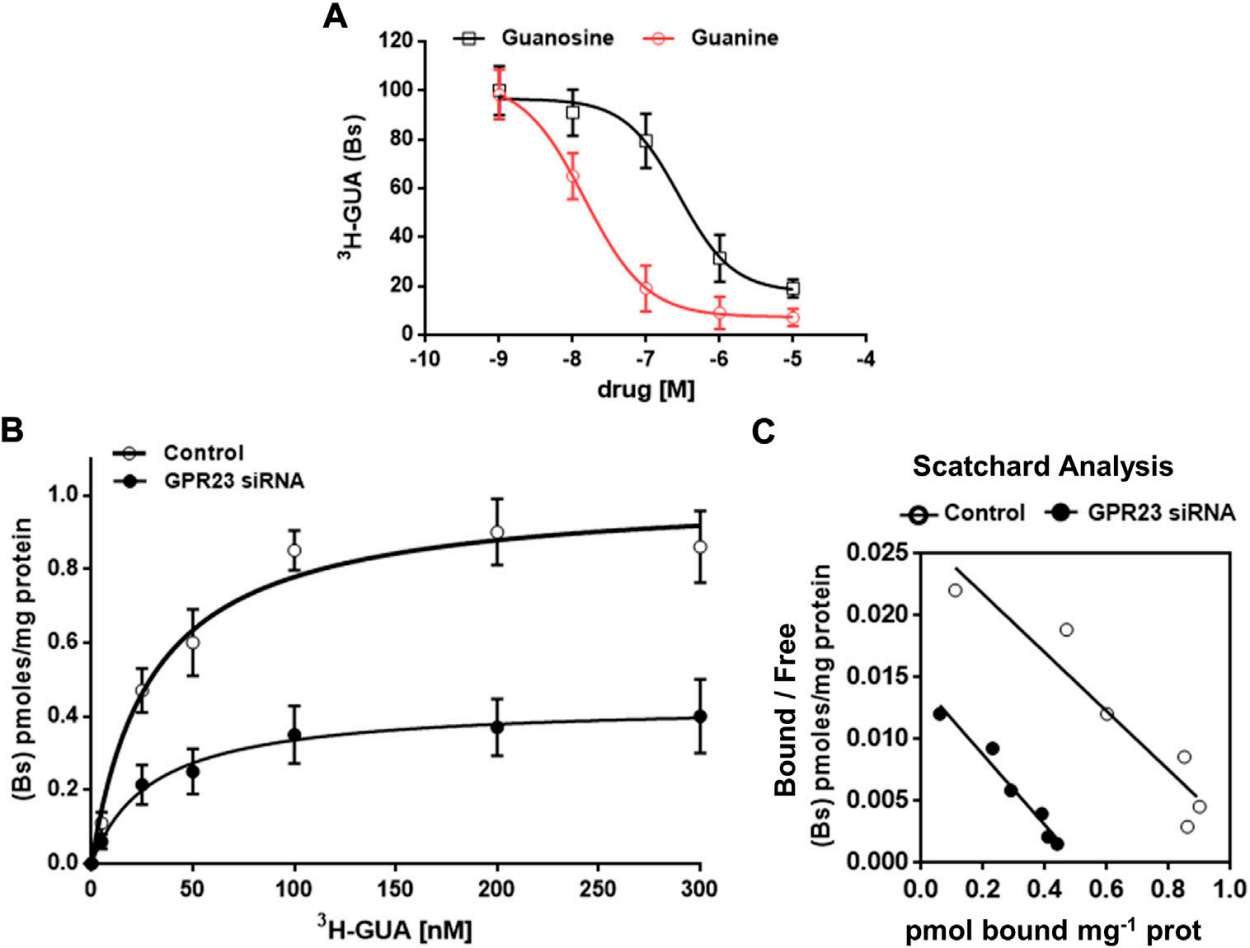
Characterization of [^3^H]-GUA radioligand binding to U87 cells membrane extracts. In all experiments membrane extracts were incubated with the indicated drugs under standard assay conditions, i.e., 30 min incubation time, 25°C temperature, pH 7.4). **(A)** Displacement of 50 nM [^3^H]-GUA binding from U87 cell membrane (50 μg protein) by unlabeled GUA or guanosine (GUO) (both at concentrations in the range from 0.001 to 10 μM). The values are the mean ± SD of three independent experiments, each point in triplicate. Statistical significance (two-way ANOVA test): ***p < 0.001 GUO vs. GUA displacement curve. **(B)** Saturation binding of [^3^H]-GUA at 25°C using control and GPR23 silenced U87 cell membranes (50 μg protein), which were incubated with increasing concentrations (6.25–300 nM) of [^3^H]-GUA under standard assay conditions. Non-specific binding was defined in the presence of 500 μM GUA. Values are the means ± SD of four experiments, each point performed in triplicate. Data in the Panel **(A,B)** were fitted by a computerized nonlinear regression analysis and resolved with a one site model. **(C)** Scatchard analysis of the data shown in **(B)**. For the panels B and C, Bs, bound specific; statistical significance (two-way ANOVA test): ***p < 0.001 vs. control.

To unequivocally demonstrate that GUA interacts with GPR23 we performed radioligand binding experiments using [^3^H]-GUA and membrane extracts from control and GPR23 silenced U87 cells. Competition binding experiments showed that GUA concentration-dependently reduced [^3^H]-GUA binding within 2 min (Ki = 0.028 ± 0.0048 µM) (Figure 6A). GUO also reduced [^3^H]-GUA binding in a concentration-dependent manner, but with much lower potency (Ki = 277.2 ± 48.4 µM). Other GBPs (GMP, GTP) tested or adenosine (ADO), even administered at concentrations up to 10 mM, showed a low percentage of [^3^H]-GUA binding with a Ki ≥ 1,000 µM (data not shown).

Additionally, we performed saturation isotherm experiments in which we observed that the binding became saturable at [^3^H]-GUA concentrations higher than 100 nM (Figure 6B). The obtained results were fitted by a computerized non-linear regression analysis and resolved for the presence of a single high affinity binding site with a K_D_ = 29.44 ± 4.07 nM and B_max_ 1.007 ± 0.035 pmol/mg prot. (Figure 6C). Importantly, when GPR23 silenced U87 cells membrane extracts were used the calculated K_D_ value was of 29.59 ± 7.57 nM, whereas B_max_ was significantly lower (0.434 ± 0.038 pmol/mg prot) (Figures 6B,C). Collectively, these results demonstrate the existence of a specific GUA binding site in membrane extracts from U87, likely being the GPR23.

## Discussion

In the present study, we identified GPR23 as a potential GPCR mediating some of the GBP-induced biological effects reported so far (Di Liberto et al., 2016; Mancinelli et al., 2020; Di Iorio et al., 2021; Massari et al., 2021). In particular, we found that GUA can interact with this receptor causing antiproliferative effects in tumoral cell lines of glioma and melanoma.

**SCHEME 1:**
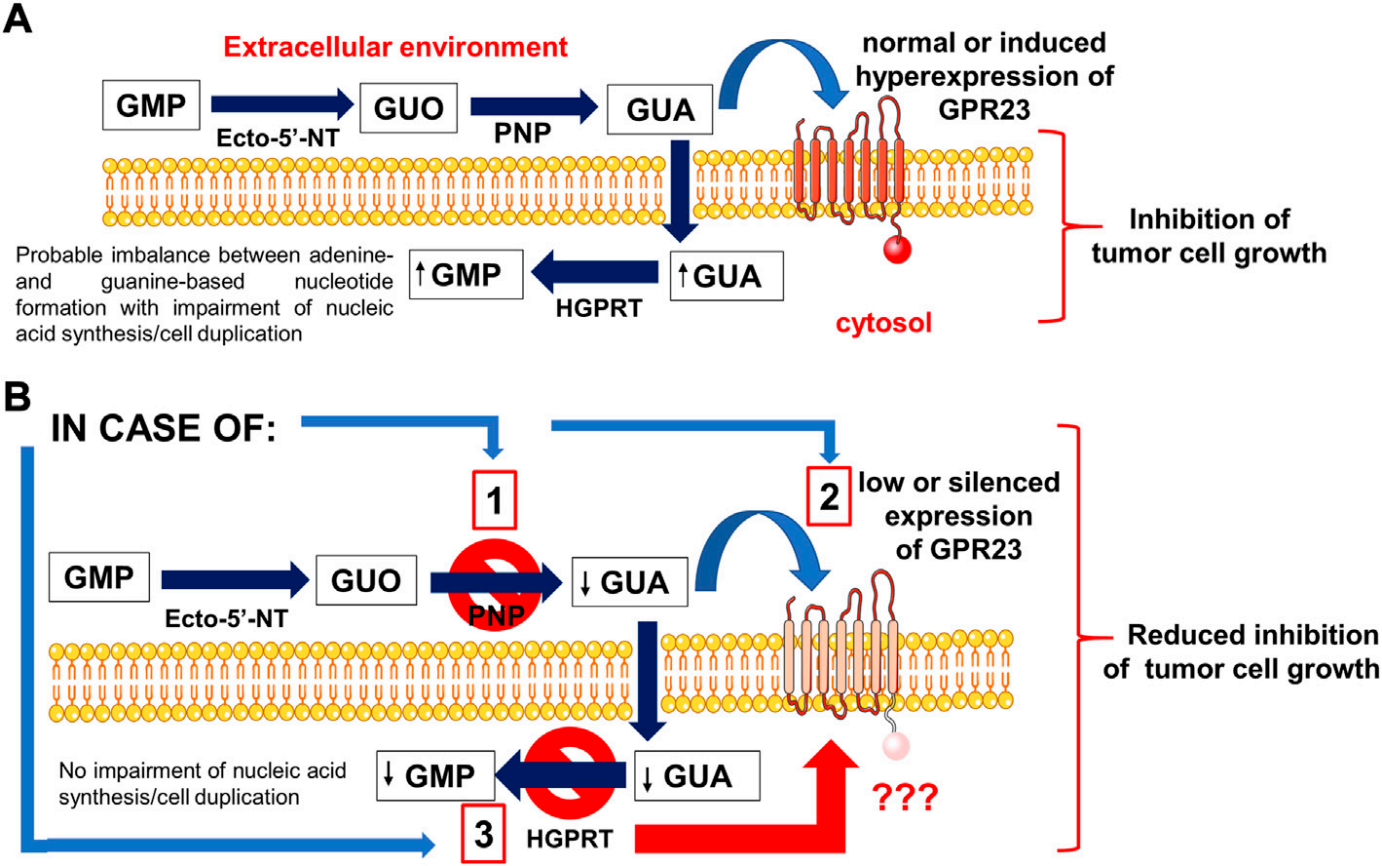
An outline of the effects promoted by GBPs, mainly GUA, in tumor cell lines. **(A)** GMP and guanosine (GUO), which are converted to guanine (GUA) by the sequential activity of the enzymes ecto-5′-nucleotidase and purine nucleoside phosphorylase (PNP), reduced the proliferation of cancer cell lines used in this study. Indeed, GUA was the actual effector of the effects induced by GMP and GUO, as the inhibition of PNP activity hindered the effects of these compounds but not that of GUA. GUA inhibited cell growth by interacting with GPR23 receptor, which was selected by a bioinformatic approach among potential candidates within a list of orphan and/or poorly characterized G-protein coupled receptors with high purinoceptor similarity. In fact, the enhanced expression of GPR23 in glioma cells or hypoxanthine-guanine phosphoribosyl-transferase (HGPRT)-mutated melanoma cell lines increased the sensitivity of these cells to GUA. **(B)** Conversely, silencing of the GPR23 receptor, as induced in glioma cells, or its low expression, as observed in melanoma cell lines with HGPRT-mutated form, reduced GUA antiproliferative effects. These findings, together with the results from binding experiments, confirmed the involvement of GPR23 in modulating responses to GUA in cancer cell lines, although further research is needed to better investigate the relationship between the activity of HGPRT and GPR23 receptor expression as well as to verify whether this orphan receptor mediates other effects of GUA.

As the first step in our research, we investigated which GPCRs, whose ligands are still unknown or poorly characterized, showed sequence similarity to P1-P2 receptors. Once selected some of them as possible candidates, i.e.; GPR3, GPR21, GPR22, GPR23, LPAR6/P2Y_5_ receptors, and after checking for their expression in cell lines, such as U87 and U873 glioma cell lines responsive to GBPs (Garozzo et al., 2010), we tested the effect of GBPs, namely GMP, GUO, and GUA, on the growth of U87 cells. We found that all GBPs decreased cell proliferation but with different efficacy and potency in their effects, GUA being the most potent compound. Furthermore, by specific siRNA silencing of the receptors aforementioned we observed that only GPR23-silencing reduced the GBP antiproliferative effects in two different glioma cell lines, mainly that induced by GUA. Additionally, GPR23-overexpressing clones, stably transfected with recombinant expression vectors, displayed an enhanced sensitivity to GUA that was reverted by siRNA-mediated silencing, thus confirming the role of GPR23 in GUA responses (see the Scheme 1).

The GPR23 gene (Accession Number NM_005296) (also called P2Y_9_ and LPAR4/P2Y_5_-like) is located on chromosome Xq13-q21.1, and contains an intronless open reading frame of 1113 bp encoding 370 amino acids (O’Dowd et al., 1997). The protein sequence shows 33% identities and 56% conserved amino acid residues vs*.* P2Y_1_ ADP receptor. Moreover, specific sites, involved in ligand interaction and conserved in P2Y protein family, are present in GPR23 protein. The consensus sequence SILFLTCIS, found in almost all functionally defined P2Y receptors (von Kügelgen and Wetter, 2000), is conserved in GPR23-protein sequence with a single substitution (SMLFLTCIS**)**. The P2Y_1_ residue Ser314, that, by mutagenesis studies, has been shown to be involved in H bond formation with N1 of purine (Jiang et al., 1997; Moro et al., 1998) is also conserved (+T301), while no residues involved in interaction with 5′-diphosphate groups are conserved (Abbracchio et al., 2003; Costanzi et al., 2004).

Further results herein reported convinced us that GPR23 could be a receptor site for GUA. In particular, we found that U87 cells released in the extracellular medium the enzyme PNP that converts GUO (also that deriving from guanine-based nucleotide metabolism) into GUA. Accordingly, cell exposure to the enzyme inhibitor, forodesine, curtailed the inhibitory effect of GMP or GUO on U87 cell growth, but not that of GUA (see the Scheme 1).

It has also to be mentioned that we previously reported (Garozzo et al., 2010) that the loss of GUA-induced antiproliferative effects in a melanoma cell line (C32TG) is related to expression of a mutated inactive form of HGPRT by these cells. Likewise, we observed a decreased potency of GUA in causing antiproliferative effects in U87 cells silenced for HGPRT transcripts. Noteworthy, the enzyme HGPRT, which catalyzes the conversion of hypoxanthine or guanine and 5-phospho-α-D-ribose 1-diphosphate (PRPP) to, respectively, inosine 5′-monophosphate (IMP) or GMP and pyrophosphate (PPi), has recently been demonstrated to be crucial in other tumoral cells such as those involved in acute myeloid leukemia (Wang et al., 2022). We have previously suggested that the excessive intracellular conversion of GUA in GMP mediated by HGPRT produced an imbalance of purine nucleotides resulting in a S-phase block of cell cycle (Garozzo et al., 2010). Accordingly, GBP recycling determined by HGPRT activity primes differentiation of these cells, reducing their growth and aggressiveness. Thus, here we wanted to ascertain if HGPRT were the only determinant factor in accounting for GUA inhibitory effects on cell growth. Our results showed that both mutated HGPRT-negative melanoma cell line as well as U87 cells, in which HGPRT has previously been silenced, expressed very low levels of GPR23. Clearly, this finding shows a close relationship between the lack of HGPRT activity and the low GPR23 expression, which has no obvious explanation and, therefore, needs to be further investigated. Anyway, the antiproliferative effects of GUA were re-established by stable transfection of a GPR23 expression construct in both cell types. Thus, as outlined in the Scheme 1, these results indicate that the metabolic mechanism linked to the purine salvage pathway could not be the only one responsible for the growth inhibitory action of GUA.

Finally, the last experiments showing the existence of a specific binding site for tritiated GUA in membrane of U87 cells add consistency to the other results so far discussed, demonstrating that GUA exerts its inhibitory effects on different tumoral cells lines, at least, by interacting with GPR23. Noteworthy, [^3^H]-GUA binding was displaced by unlabelled GUO, but at concentrations higher than those of unlabelled GUA, suggesting that GUO could also bind GPR23 but with lower affinity. Clearly, this aspect deserves further investigation. In contrast, guanine-based nucleotides or ADO displaced tritiated GUA at so high concentrations which are usually considered as not consistent with a selective binding of these compounds to a receptor, GPR23 in our case.

Regarding specific ligands and biological functions of GPR23, previous functional studies reported that is activated by LPA (Noguchi et al., 2003; Lee et al., 2007; Yanagida et al., 2007). For this compound different receptor isoforms (LPA_1-6_) have been recognized, which are distinguished into isoforms belonging to the endothelial differentiating gene (Edg) family of GPCR and non-Edg family of purinergic receptors. Among GPCRs, three receptors for LPA have been identified, termed LPA1, LPA2, and LPA3 (previously Edg2, Edg4 and Edg7, respectively), with LPA1/Edg2 being the first identified and most widely expressed subtype. LPA1, LPA2 and LPA3 receptors are found in many cell types, exhibit relatively close homology with each other and show a high affinity for LPA (in the nanomolar range). In contrast, LPA_4_ (GPR23/P2Y_9_), LPA_5_ (GPR92), and LPA_6_ (P2RY_5_) belong to a non-Edg subfamily of purinergic GPCR clusters (reviewed in Meduri et al., 2021). Since GPR23 has been suggested to be a fourth receptor for LPA, structurally different from EDG receptors, it could not be excluded that the decrease of the GUA-induced antiproliferative effects observed following GPR23 silencing were a consequence of a decreased LPA-induced activity of this receptor. However, the basal LPA concentration in the culture medium, due to the serum supplementation, is probably too low (0.1 µM) to induce receptor activation. Clearly, future investigation will address whether and how LPA, besides GUA, might modify tumor cell growth interacting with the same receptor, maybe on different sites. About this possibility, Wetter et al. (2009) have reported that unknown serum-born compounds can activate GPR23 or potentiate the response of this receptor to LPA. On the other hand, the notion that LPA represents the main agonist of GPR23 has been challenged by Yin et al. (2009) that, using a GPCR assay that measures beta-arrestin binding to GPCRs, did not observe any response of GPR23 to LPA up to 100 µM.

In conclusion, findings herein reported indicate that GPR23 protein can represent a membrane receptor responsive to extracellular GUA, although the existence of intracellular mechanisms, even HGPRT-independent and potentiated by GPR23 expression could be an alternative explanation that cannot be ruled out. On the other hand, it has been reported that isolated basolateral membranes of renal proximal tubule express a Gi protein-coupled receptor for GUA, whose stimulation inhibits the activity of Na^+^/K^+^-ATPase (Wengert et al., 2011). To clarify these aspects, further experiments are necessary, which will allow to better characterize the modality through which GUA acts as well as to define the role played by GPR23 as the membrane receptor mediating the extracellular activity of GUA.

## Data Availability

The gene expression datasets used in this study can be found in Supplementary Material and online repositories, whose link are reported in the article.
