# Supplementary figures and images for: Guanine inhibits the growth of human glioma and melanoma cell lines by interacting with GPR23
Source: Front Pharmacol. 2022 Sep 19;13:970891. doi: 10.3389/fphar.2022.970891 (PMC9527276; doi:10.3389/fphar.2022.970891)

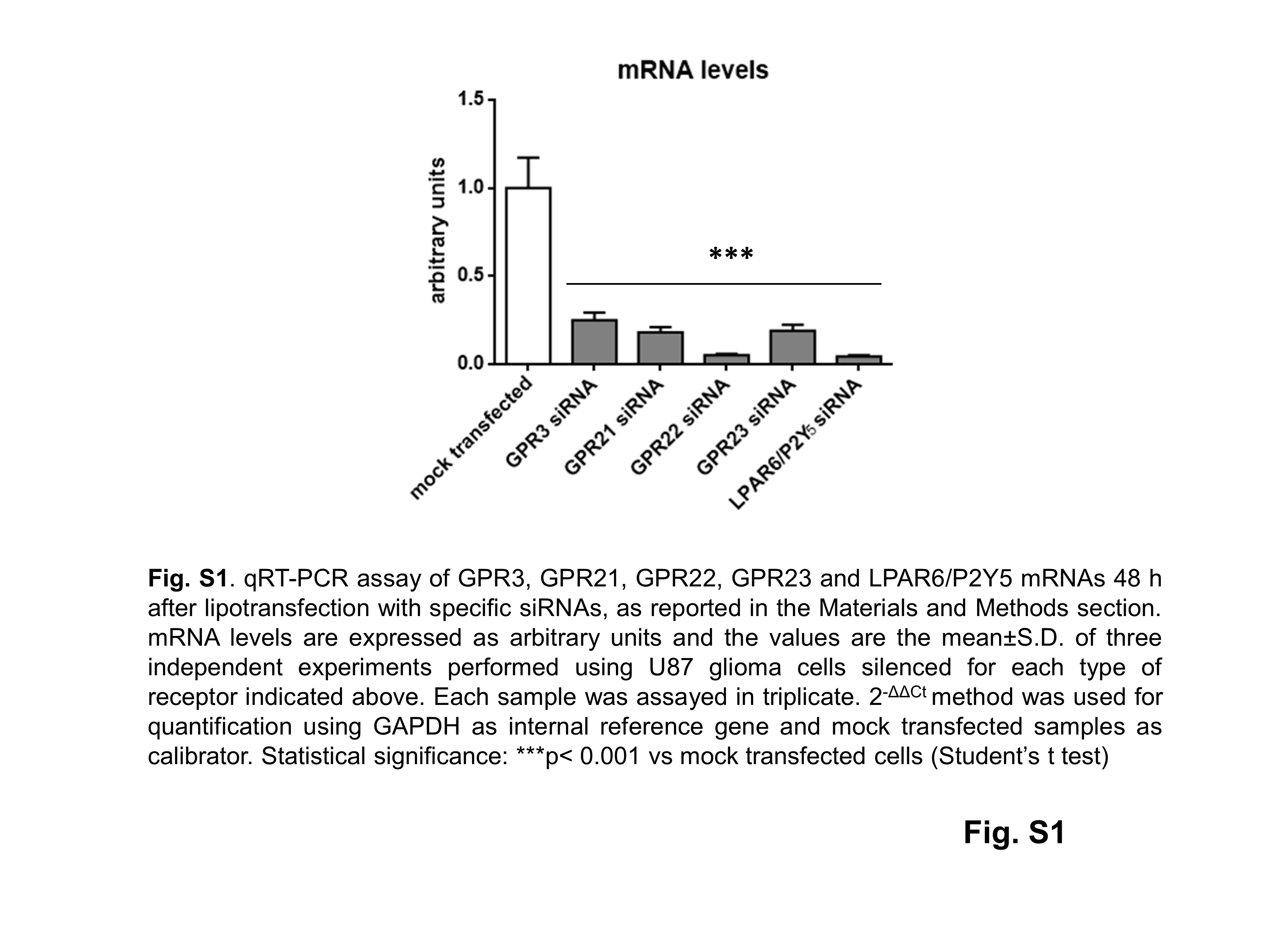

Supplement: Supplementary file 1 [file Image1.TIF]
