# Supplementary material for: Guanine inhibits the growth of human glioma and melanoma cell lines by interacting with GPR23
Source: Front Pharmacol. 2022 Sep 19;13:970891. doi: 10.3389/fphar.2022.970891 (PMC9527276; doi:10.3389/fphar.2022.970891)
Supplement: Supplementary file 3 [file DataSheet1.docx]

**Table S1: specific primers corresponding to the nucleotide sequences of the indicated G-protein coupled receptors (GPCRs) used for the identification of the respective mRNA expression by Polymerase Chain Reaction (qualitative PCR)**

| **Receptor**  **name** | **GenBank**  **Accession**  **number** | **Sequence** |
| --- | --- | --- |
| **GPR3** | NM_005281 | Fw: 5’-GCTCAGGCAACGTGAATGTA-3’;  Rv: 5’-GATGGTTCTTGGAGAGTGGA-3’; |
| **GPR6** | NM_005284 | Fw: 5’-ACGCGCTCACCTATTACTCG-3’;  Rv: 5’-GCGGAAGGCATAGATGATGG-3’ |
| **GPR12** | NM_005288 | Fw: 5’-CTACCTCTCACTGTACTACG-3’;  Rv: 5’-CTGGATGTAGAGCTGAAGCA-3’ |
| **GPR20** | NM_005293 | Fw: 5’-CTGACTGTCCTGGAGTTCCT-3’;  Rv: 5’-GACGAAGCAGTAGACGATGG-3’ |
| **GPR21** | NM_005294 | Fw: 5’-GAGTCCTTGACTTGCCAGAT-3’;  Rv: 5’-ATCTGGCAAGTCAAGGACTC-3’ |
| **GPR22** | NM_005295 | Fw: 5’-ACCACACAACATGAGGCTAC-3’;  Rv: 5’-ATTGGTGTCCAGCAGAGAAG-3’ |
| **GPR23** | NM_005296 | Fw: 5’-CCGGTCCATAGTGTCAGAGT-3’;  Rv: 5’-ACTAGGATCCAGACACCAGC-3’ |
| **LPAR6/P2Y5** | NM_005767) | Fw: 5'-CGATGGTAAGCGTTAACAGC-3';  Rv: 5'-ACAGTTAACCACACGCCAGT-3' |
| **P2Y8** | NM_178129 | Fw: 5’-CGGCCGTCGACTTAAGCGTT-3’;  Rv: 5’-CCACTTGAGGACGTCGAAGC-3’ |
| **P2Y10** | NM_014499 | Fw: 5'-GATCCATGCCGCCAGAGTAA-3';  Rv: 5'-TGATGAAGCGGCACAGAACC-3' |
| **GPR174** | NM_032553 | Fw: 5'-ACTGTCATTCTTGTGCCAGG-3';  Rv: 5'-TAGGACAATCAGAAGCGGAG-3' |
| **GPR101** | NM0_54021 | Fw: 5'-GTTATCTTCCTCGCCGCCTC-3';  Rv: 5'-CGATGAAGGACACCACGCTG-3' |
| **EB12** | NM_004951 | Fw: 5'-AGCACGGCCAGGATAGTAAT-3';  Rv: 5'-TGAAGCGGTCAATACTCAGG-3' |
| **TAR3** | AF_380189 | Fw: 5'-TGGCCTGTGCTGACTTCTTG-3';  Rv:5'-TCTGAGGAGGACTGAGCTTG-3' |
| **GPR161** | NM_007369 | Fw: 5'-AGCACCTCCACCTCCTCTTC-3';  Rv: 5'-CACGCAGGTTACCTGAGTCC-3' |
| **GPR52** | NM_005684 | Fw: 5'-CCACAGGTGTCCACGAGTCA-3';  Rv: 5'-ATGGTGTAGCGACGGTCAGG-3' |
| **GPR159** | NM_123456 | Fw: 5'-AACAGCAGCGACTGCATCGT-3';  Rv:5'-TGCCGAAGAGGTTGATGGAG-3' |
| **GPR34** | NM_05300 | Fw: 5'-ACCAAGACGAGTCTCAGTGT-3';  Rv: 5'-GAGGCAGAAGATGAGTAGGA-3' |
| **LPA/1 var1/2** | NM_001401/  NM_057159 | Fw: 5'-GTAGTGGTGGTCATTGTGGT-3';  Rv: 5'-GCTGGTTCCTCATCTCAGTT-3' |
| **LPA2** | NM_004720 | Fw: 5'-AGCCTGGTCAAGACTGTTGT-3';  Rv: 5'-GGCAGAGGATGTATAGTGGA-3' |
| **LPA3** | NM_012152 | Fw: 5'-AACATCTCTGCCTGCTCTTC-3';  Rv: 5'-CCATACATGTCCTCGTCCTT-3' |

Abbreviations: Fw, forward; Rv, reverse

**Table S2: Fw (forward), reverse (Rv) and fluorigenic primers used for quantitative real-time RT-PCR.** For each GPCR mRNA expression, three sequence-specific oligonucleotides were designed using the Primer Express oligo design software (Applied Biosystem, Carslbad, CA, USA). Two of them were forward (Fw) and Reverse (Rv) primers used for PCR amplification. The third sequence (TaqMan Probe, Applied Biosystem, Carslbad, USA) was a fluorogenic probe labelled with a fluorescent reporter dye (6-FAM) and a quencher dye (TAMRA) attached at the 5’and 3’ end, respectively. The probe was designed to hybridize the portion of PCR amplicon between Fw and Rv primers.

| **Receptor**  **name** | **Primer sequence** | **Fluorogenic probe** |
| --- | --- | --- |
| **GPR3** | Fw: 5’-GGCCTTTACCGCCAGCAT-3’  Rv: 5-CGTGTCACTGTTGTCTCTGAATA-3’ | 5’-TCACTGTCGACCGCTACCTTTCTCTGTACAAT-  TAMRA 3’ |
| **GPR21** | Fw: 5’-GGCAAACCTGGATATCATGGA-3’  Rv: 5’-ACGATGAACAGGGTGAAGTAGGA-3’ | 5’-TTTCAGTGGTGTGCGGAGTCCTGG-TAMRA 3’ |
| **GPR22** | Fw: 5’-ATGAGGGCTTGTGTATCTTTTGCAA-3’  Rv: 5’-GCAGGTTTTACAGAGATGTCATATCT- 3’ | 5’-TGTCTCAACAGCAATCAACGTTTTTGCTATCA-TAMRA 3’ |
| **GPR23** | Fw: 5’-ATATTTCCTACCGGTCCATAGTG- 3’  Rv: 5’-TGGAAGTCAA TGAATCTTCT GTCA- 3’ | 5’-CAGAGTGGTG AACCCCTGCA G- TAMRA 3’ |
| **LPAR6/**  **P2Y5** | Fw: 5’-TCTTGTTGAATTGAGGCCTTTT-3’  Rv:5’-AAACATGTGAGAATCATCTACAAAGA-3’ | 5’-CCAAGAATTGTGAGAGAAAGCGACCTC-  TAMRA 3’ |

**Table S3: siRNAs primers used for silencing GPR3, GPR21, GPR22, GPR23, LPAR6/P2Y_5_ receptors and the enzyme hypoxanthine-guanine phosphoribosyl transferase (HGPRT).** The sequences were subjected to BLAST search to confirm the absence of homology to other additional known coding sequences in the genome human project. siRNAs were chemically synthesized by MWG Biotech AG (Ebersberg, Germany) and resuspended according to the manufacturer’s instructions. RNA not complementary to any cellular transcript (ctrlRNA) was used as control.

| **Receptor submitted to siRNA** | **Primer sequence** |
| --- | --- |
| **GPR3-siRNA** | sense sequence: 5’-UGGCCUUUACCGCCAGCAU-dTT-3’  antisense sequence: 5’-AUGCUGGCGGUAAAGGCCA- dTT-3’ |
| **GPR21-siRNA** | sense sequence: 5’-CCUGGAUAUCAUGGAGAUG- dTT-3’  antisense sequence: 5’-CAUCUCCAUGAUAUCCAGG-dTT-3’ |
| **GPR22-siRNA** | sense sequence: 5’-GUGUCUCAACAGCAAUCAA- dTT-3’  antisense sequence: 5’-UUGAUUGCUGUUGAGACAC- dTT-3’ |
| **GPR23-siRNA** | sense sequence: 5’-GAUCUCUGGAACUGCAUUC-dTT-3’  antisense sequence: 5’-GAAUGCAGUUCCAGAGAUC- dTT-3’ |
| **LPAR6/P2Y_5_-siRNA** | sense sequence: 5’-CCUGUUACAUUAAGUAGAA-dTT-3’  antisense sequence: 5’-UUCUACUUAAUGUAACAGG-dTT-3’ |
| **HGPRT** | sense sequence: 5’-GUAAUUGGUGGAGAUGAUCdTdT-3’  antisense sequence: 3’-dTdTCAUUAACCACCUCUACUAG-5’ |
| **ctrlRNA** | sense sequence: 5’-CUAGAUCGCUACGAAGUAU-dTT-3’  antisense sequence: 5’-AUACUUCGUAGCGAUCUAG-dTT-3’ |
